# Characterization of the complete plastome sequence of *Torreya grandis* var. *jiulongshanensis* (Taxaceae), a rare and endangered plant species endemic to Zhejiang province, China

**DOI:** 10.1080/23802359.2020.1715895

**Published:** 2020-01-24

**Authors:** Ming Jiang, Junfeng Wang, Huijuan Zhang

**Affiliations:** a Zhejiang Provincial Key Laboratory of Plant Evolutionary and Conservation, College of Life Sciences, Taizhou University, Jiaojiang, China;; b Lishui Institute of Forestry, Lishui, China

**Keywords:** *Torreya grandis* var. *jiulongshanensis*, plastome, phylogenetic analysis, rare plant species

## Abstract

*Torreya grandis* var. *jiulongshanensis* (Taxaceae) is an evergreen tree endemic to Zhejiang province, China, and it is listed as a rare plant species with extremely small populations. In our present study, we assembled the chloroplast (CP) genome of *T. grandis* var. *jiulongshanensis* using high-throughput sequencing data generated by an Illumina Hiseq X Ten platform. The results indicate that the complete CP genome is 136,705 bp in size with an overall guanine and cytosine content of 35.5%. The plastome has lost its quadripartite structure. In the CP genome, there are a total of 119 genes, including 82 protein-coding genes, 4 ribosomal RNA genes, and 33 transfer RNA genes. Phylogenetic analysis revealed that *T. grandis* var. *jiulongshanensis* is a sister species to *T. grandis*.

*Torreya grandis* var. *jiulongshanensis* is an evergreen tree with gray bark and leafy branchlets (Wu et al. 1999). The plant species occurs only in counties of Suichang, Jinyun, Songyang, and Tiantai, Zhejiang province, China. In the wild, *T. grandis* var. *jiulongshanensis* has fewer than 100 individuals, and it is described as ‘plant species with extremely small populations’. The chloroplast (CP) genome sequences of three *Torreya* species, *T. grandis*, *T. parvifolia*, and *T. fargesii*, were assembled. However, the CP genome of *T. grandis* var. *jiulongshanensis* has not been reported. Herein, we assembled and annotated the CP chloroplast genome of *T. grandis* var. *jiulongshanensis*, a plant species morphologically similar to *T. grandis*.

Leaves were sampled near Xikengxia Village (28°19.37′N, 118°55.53′E), Jingning She Autonomous County, Zhejiang, China. A voucher specimen coded CHS2017103 was deposited at the Molecular Biology Laboratory at the Taizhou University. Total genomic DNA was extracted according to the CTAB protocol (Doyle and Doyle, 1987). A DNA library was constructed following the manufacturer’s instructions, and was then sequenced to obtain raw data on an Illumina Hiseq X Ten platform (Illumina, CA, USA). Totally, 5.5 Gb of clean data were generated, and the reads were applied to assemble the CP genome by NOVOPlasty (Dierckxsens et al. 2017). The complete plastome was annotated using Dual Organellar GenoMe Annotator (DOGMA), and the gene boundaries were adjusted manually (Wyman et al. 2004). The programs, namely tRNAscan-SE and ARAGORN, were applied to detect tRNAs in the CP genome (Lowe and Eddy 1997; Laslett and Canback 2004). The *T. grandis* var. *jiulongshanensis* CP genome (GenBank accession: MN527333) is 136,705 bp long with an overall guanine and cytosine content of 35.5%, and it composes of 32.5% adenine, 17.7% cytosine, 17.9% guanine, and 32.0% thymine.

Typically, the CP genome is a circular molecule with a quadripartite structure, comprising a small single-copy and a large single-copy separated by a pair of inverted repeats (Khan et al. 2017). However, the plastome of *T. grandis* var. *jiulongshanensis* has lost its quadripartite structure. The genome consists of 119 genes, including 82 protein-coding genes, 4 ribosomal RNA genes, and 33 transfer RNA genes. Among them, 15 genes (*atpF*, *ndhA*, *ndhB*, *petB*, *petD*, *rpl2*, *rpl16*, *rpoC1*, *rps12*, *rps16*, *trnG-UCC*, *trnI-GAU*, *trnK-UUU*, *trnL-UAA*, and *trnV-UAC*) possess one intron, while another two genes (*rps12* and *ycf3*) contain two introns.

To complete the CP genome sequences of *Amentotaxus argotaenia* (KR780582), *A. formosana* (AP014574), *Cephalotaxus hainanensis* (NC_042392), *Taxus brevifolia* (NC_041502), *T. chinensis* (NC_041496), *T. floridana* (MH390474), *T. globose* (MH390488), *T. fargesii* (NC_029398), *T. grandis* (NC_034806), *T. parvifolia* (NC_043866), *Thuja_occidentalis* (NC_042177), five *Pinus*, as well as six *Araucuria* species were retrieved from GenBank, and a phylogenetic tree was generated by PhyML 3.1 software under the GTR + G + I model (Guindon et al. 2010). The 27 plants can be divided into 11 different subgroups, namely *Amentotaxus* subgroup, *Pseudotaxus* subgroup, *Taxus* subgroup, and *Torreya* subgroup, etc. Our results indicated that *T. grandis* var. *jiulongshanensis* was sister to the three *Torreya* species, with a support value of 100% (Figure 1).

**Figure 1.**
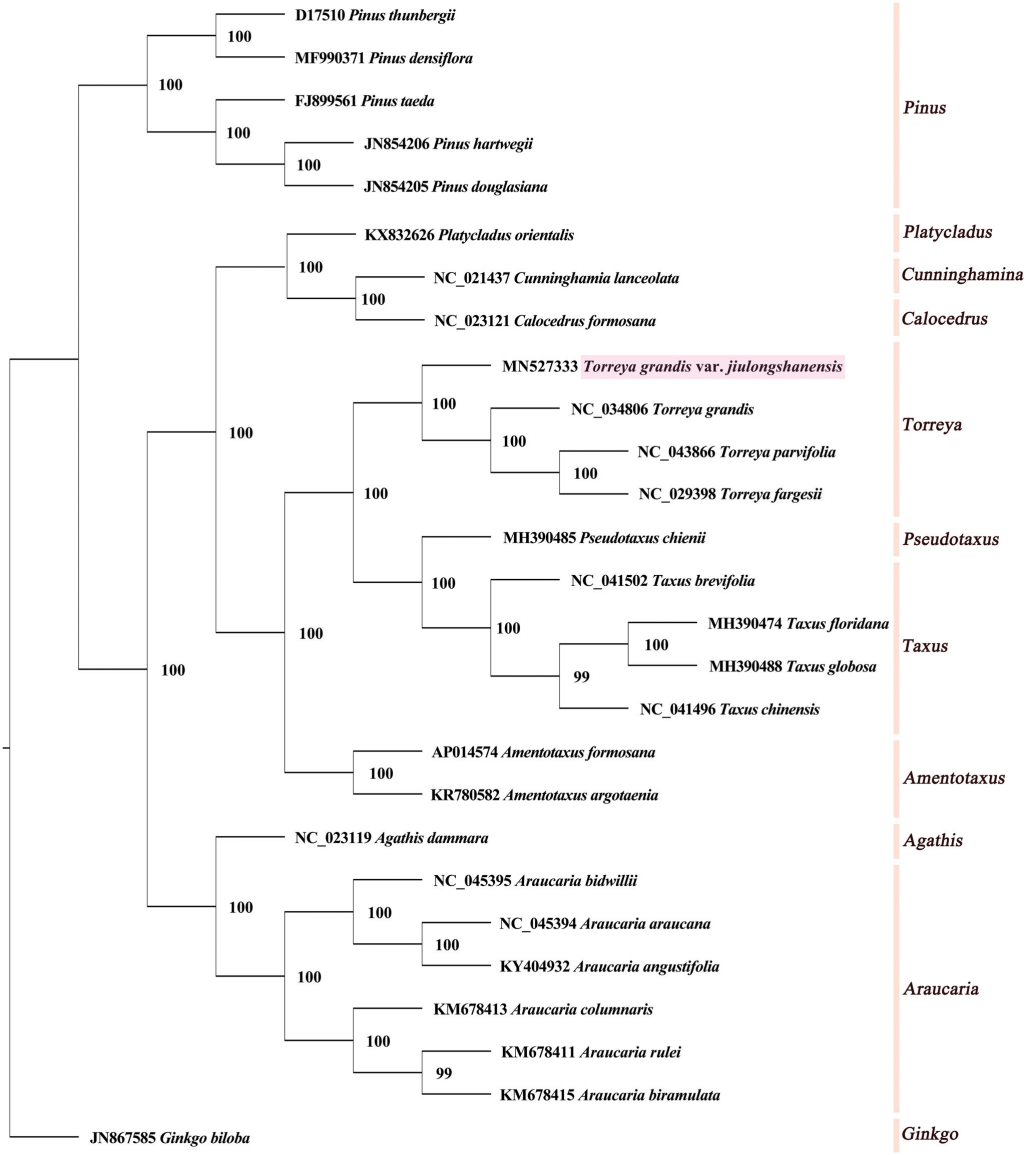
Maximum likelihood tree of 27 plant species based on chloroplast genome sequences, with *Ginkgo biloba* (JN867585) as the outgroup. The numbers next to nodes indicate bootstrap support values.

## References

[CIT0001] Dierckxsens N, Mardulyn P, Smits G 2017 NOVOPlasty: de novo assembly of organelle genomes from whole genome data. Nucleic Acids Res. 45(4):e18. 28204566 10.1093/nar/gkw955 PMC5389512

[CIT0002] Doyle JJ, Doyle JL 1987 A rapid DNA isolation procedure for small quantities of fresh leaf tissue. Phytochem Bull. 19:11–15.

[CIT0003] Guindon S, Dufayard JF, Lefort V, Anisimova M, Hordijk W, Gascuel O 2010 New algorithms and methods to estimate maximum-likelihood phylogenies: assessing the performance of PhyML 3.0. Syst Biol. 59(3):307–321. 20525638 10.1093/sysbio/syq010

[CIT0004] Khan AL, Al-Harrasi A, Asaf S, Park CE, Park GS, Khan AR, Lee IJ, Al-Rawahi A, Shin JH 2017 The first chloroplast genome sequence of *Boswellia sacra*, a resin-producing plant in Oman. PLoS One. 12(1):e0169794. 28085925 10.1371/journal.pone.0169794 PMC5235384

[CIT0005] Laslett D, Canback B 2004 ARAGORN, a program to detect tRNA genes and tmRNA genes in nucleotide sequences. Nucleic Acids Res. 32(1):11–16. 14704338 10.1093/nar/gkh152 PMC373265

[CIT0006] Lowe T M, Eddy S R 1997 tRNAscan-SE: a program for improved detection of transfer RNA genes in genomic sequence. Nucleic Acids Res. 25(5):955–964. 9023104 10.1093/nar/25.5.955 PMC146525

[CIT0007] Wu ZY, Raven PH, Hong DY 1999 Flora of China. Vol. 4, Beijing: Science Press; St. Louis: Missouri Botanical Garden Press.

[CIT0008] Wyman SK, Jansen RK, Boore JL 2004 Automatic annotation of organellar genomes with DOGMA. Bioinformatics. 20(17):3252–3255. 15180927 10.1093/bioinformatics/bth352

